# Financing of Pharmaceutical Services in the municipal management of the Brazilian Unified Health System

**DOI:** 10.11606/S1518-8787.2017051007060

**Published:** 2017-09-22

**Authors:** Daniel Resende Faleiros, Francisco de Assis Acurcio, Juliana Álvares, Renata Cristina Rezende Macedo do Nascimento, Ediná Alves Costa, Ione Aquemi Guibu, Orlando Mario Soeiro, Silvana Nair Leite, Margô Gomes de Oliveira Karnikowski, Karen Sarmento Costa, Augusto Afonso Guerra

**Affiliations:** I Programa de Pós-Graduação em Medicamentos e Assistência Farmacêutica. Faculdade de Farmácia. Universidade Federal de Minas Gerais. Belo Horizonte, MG, Brasil; II Departamento de Farmácia Social. Faculdade de Farmácia. Universidade Federal de Minas Gerais. Belo Horizonte, MG, Brasil; III Instituto de Saúde Coletiva. Universidade Federal da Bahia. Salvador, BA, Brasil; IV Departamento de Saúde Coletiva. Faculdade de Ciências Médicas. Santa Casa de São Paulo. São Paulo, SP, Brasil; V Faculdade de Ciências Farmacêuticas. Pontifícia Universidade Católica de Campinas. Campinas, SP, Brasil; VI Departamento de Ciências Farmacêuticas. Universidade Federal de Santa Catarina. Florianópolis, SC, Brasil; VII Faculdade de Ceilândia. Universidade de Brasília. Brasília, DF, Brasil; VIII Núcleo de Estudos de Políticas Públicas. Universidade Estadual de Campinas. Campinas, SP, Brasil

**Keywords:** Pharmaceutical Services, economics, Healthcare Financing, Primary Health Care, Health Services Research, Unified Health System, Assistência Farmacêutica, economia, Financiamento da Assistência à Saúde, Atenção Primária à Saúde, Pesquisa sobre Serviços de Saúde, Sistema Único de Saúde

## Abstract

**OBJECTIVE:**

To discuss factors related to the financing of the Basic Component of Pharmaceutical Services within the municipal management of the Brazilian Unified Health System.

**METHODS:**

The *Pesquisa Nacional sobre Acesso, Utilização e Promoção do Uso Racional de Medicamentos no Brasil – Serviços* (PNAUM – National Survey on Access, Use and Promotion of Rational Use of Medicines – Services) is a cross-sectional, exploratory, and evaluative study that performed an information survey in a representative sample, stratified by Brazilian regions It considered different study populations in the sampling plan, which represent primary health care services in the cities. Data were collected in 2015 by two methods: in person, by applying direct observation scripts and interviews with users, physicians, and professionals responsible for the dispensing of medicines in primary care services; by telephone interviews with municipal health managers and municipal professionals responsible for Pharmaceutical Services. The results were extracted from the questionnaires applied by telephone.

**RESULTS:**

Of the sample of 600 eligible cities, we collected 369 interviews (61.5%) with secretaries and 507 (84.5%) with pharmaceutical services managers. 70.8% of the cities have a computerized management system; and 11.9% have qualification/training of professionals. More than half (51.3%) of the cities received funds for the structuring of pharmaceutical services, and almost 60% of these cities performed this type of spending. In 35.4% of cases, municipal secretaries of health said that they use resources of medicines from the *Componente Básico da Assistência Farmacêutica* (CBAF – Basic Component of Pharmaceutical Services) to cover demands of other medicines, but only 9.7% believed that these funds were sufficient to cover the demands. The existence of a permanent bidding committee exclusively for acquiring medicines was reported in 40.0% of the cities.

**CONCLUSIONS:**

We found serious deficiencies in the public financing of medicines, as well as little concern about the formality in the use of public resources, expenses that meet individual demands to the detriment of the community, insufficient resources allocated to the Basic Component of Pharmaceutical Services, and exhaustion of the financing model.

## RESUMO

**OBJETIVO:**

Discutir fatores relacionados ao financiamento do Componente Básico da Assistência Farmacêutica no âmbito da gestão municipal do Sistema Único de Saúde.

**MÉTODOS:**

A Pesquisa Nacional sobre Acesso, Utilização e Promoção do Uso Racional de Medicamentos no Brasil – Serviços é um estudo transversal, exploratório, de natureza avaliativa, que realizou levantamento de dados em amostra estratificada pelas regiões brasileiras, consideradas diferentes populações de estudo no plano de amostragem representativa de serviços de atenção primária em municípios. Os dados foram coletados em 2015, na forma presencial mediante a aplicação de roteiros de observação direta, entrevistas com usuários, médicos e responsáveis pela entrega dos medicamentos nos serviços de atenção primária e entrevistas telefônicas com gestores municipais de saúde e responsáveis municipais pela assistência farmacêutica. Os resultados foram extraídos dos questionários aplicados por telefone.

**RESULTADOS:**

Da amostra de 600 municípios elegíveis, foram coletadas 369 entrevistas (61,5%) com secretários e 507 (84,5%) com responsáveis pela assistência farmacêutica. Em 70,8% dos municípios existe sistema informatizado de gestão; e em 11,9% qualificação/capacitação dos profissionais. Mais da metade (51,3%) dos municípios receberam recursos destinados à estruturação da assistência farmacêutica, quase 60% desses municípios realizou este tipo de gastos. Em 35,4% dos casos, secretários municipais de saúde afirmaram utilizar recursos de medicamentos do Componente Básico da Assistência Farmacêutica para cobrir demandas de outros medicamentos, mas apenas 9,7% acreditam que esses recursos são suficientes para atender à demanda. A existência de comissão permanente de licitação exclusiva para a aquisição de medicamentos foi registrada em 40,0% dos municípios.

**CONCLUSÕES:**

São graves as deficiências, a pouca preocupação com a formalidade na execução dos recursos públicos, os gastos ocorridos para atendimento de demandas individuais em detrimento da coletividade, a insuficiência de recursos destinados ao Componente Básico da Assistência Farmacêutica e a exaustão do modelo de financiamento.

## INTRODUCTION

The financing of health systems has become a major challenge before the growing demand for health actions and services. The pharmaceutical spending has been settling as a real threat to the sustainability of public systems^16^. These expenses can be quite significant because of the high unit value of some medicines or by the large volume of items needed to meet the needs of the population.

One way or another, these values are expressive before the set of total spending on health care, either on the account of citizens or Government. In 1996, medicines accounted for 37% of the total spending of Brazilian families with health, becoming 47% in 2008^10^. On the other hand, the spending forecast of the Brazilian Unified Health System (SUS) for the promotion and organization of Pharmaceutical Services (PS); maintenance and operation of the Popular Pharmacy Program (free and co-payment); production of pharmaceuticals, medicines, and herbal medicines; acquisition and distribution of medicines of PS Components and for the treatment of HIV/AIDS and other sexually transmitted diseases totaled R$ 13.2 million for 2017, representing more than 11% of the total expenditures set out for the Brazilian Ministry of Health^7^.

The *Pesquisa Nacional Sobre Acesso, Utilização e Promoção do Uso Racional de Medicamentos – Serviços* (PNAUM – National Survey on Access, Use and Promotion of Rational Use of Medicines – Services)^11^ aimed to characterize the organization of PS in SUS primary health care, focusing the promotion of access and rational use of medicines, as well as the identification and discussion of factors that compromise the consolidation of the PS in the cities. This study is part of PNAUM – Services and aimed to discuss factors related to the financing of the *Componente Básico da Assistência Farmacêutica* (CBAF – Basic Component of Pharmaceutical Services) in Brazilian cities.

## METHODS

PNAUM – Services is a cross-sectional, exploratory, and evaluative study, consisting of a information survey in a representative sample of primary health care services in cities. The sample was stratified by the Brazilian regions, considering the different study populations in the sampling plan^1^.

Data were collected from July 2014 to May 2015, by face-to-face and telephone interviews. In person, data were collected by applying direct observation scripts, interviews with patients, physicians, and professionals responsible for the dispensing of medicines in SUS primary health care services. By phone, data were collected using semi-structured questionnaires for municipal health managers and professionals responsible for PS in the cities, applied by interviews conducted by trained researchers, with computer-assisted telephone interview technology. All interviews, in-person or by telephone, were preceded by the signing of the informed consent form^1^.

The instruments aimed to know the organization of PS, including their financing and the local conduct on the use of financial resources destined to the CBAF. The results relevant to finance-related factors were extracted of the questionnaires applied to municipal secretaries of health and those responsible for the PS of the cities, listed in the following groups of variables: management of financial resources, use of computerized systems, professional training and qualification, ways of acquiring medicines and structuring PS.

Statistical analysis included estimates of absolute and relative frequencies (with 95% confidence intervals). Group comparison was held by Pearson’s Chi-square test. Data analysis was performed with SPSS^®^ software, version 22, using the plan of complex samples^1^.

The research project was approved by the National Research Ethics Committee, by Opinion no. 398,131, September 16, 2013.

## RESULTS

Of the calculated sample of 600 cities eligible for the study, 369 interviews were conducted (61.5%) with municipal secretaries of health and 507 (84.5%) with those responsible for PS.

**Figure 1:**
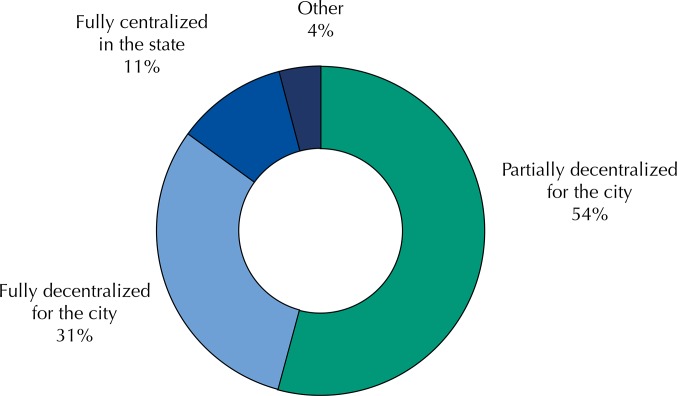
Agreement form for the use of resources of the Basic Component of Pharmaceutical Services (CBAF) in the cities, according to municipal secretaries of health. National Survey on Access, Use and Promotion of Rational Use of Medicines – Services, 2015.

According to the secretaries, 54% of Brazilian cities have adopted the partial decentralization regime agreeing with the implementation of CBAF resources, followed by 30.8% who have adopted the fully decentralized regime (Figure 1). The PS coordination had full autonomy of management of the financial resources defined for PS in 22.7% of the cities, and partial in 45.6%. Of the cities evaluated, 67.3% made expenses intended for the structuring of PS in the year preceding the research. CBAF resources were used to cover demands for medicines not belonging to this component by 35.4% of the cities. 9.7% of municipal secretaries of health stated that the resources intended for CBAF were sufficient to meet the demands of the population.

PS: Pharmaceutical Services

Source: PNAUM – Services, 2015.

From the perspective of those responsible for PS, the PS coordination had full autonomy for managing financial resources in 19.7% of the cities, and partial autonomy in 38.2%. They reported that 70.8% of Brazilian cities had a computerized system for PS management, especially in the South region (88.8%). Activities for qualification and/or professional training for workers of PS occurred in 11.9% of the cities (Table 1).

**Table 1:** Data reported by those responsible for municipal Pharmaceutical Services, according to Brazilian regions. National Survey on Access, Use and Promotion of Rational Use of Medicines – Services, 2015.

Variable	Midwest		North		Northeast		South		Southeast		Brazil	
					
	n^a^	% (95%CI)	n^a^	% (95%CI)	n^a^	% (95%CI)	n^a^	% (95%CI)	n^a^	% (95%CI)	n^a^	% (95%CI)
The coordination of PS has autonomy in financial management												
Yes, fully	17	19.5 (12.4–29.2)	10	10.8 (5.6–19.6)	16	15.3 (8.7–25.3)	24	24.3 (16.7–33.9)	20	22.8 (15–33)	87	19.7 (15.7–24.4)
Yes, partially	30	30.9 (22.2–41.3)	22	25 (16.7–35.6)	35	38.4 (28–49.9)	43	42.3 (32.8–52.5)	38	39.8 (29.9–50.7)	168	38.2 (33–43.6)
Existence of a computerized system for the management of PS^b^												
Yes	54	53.5 (43.5–63.3)	41	40.1 (30.5–50.5)	60	61.2 (50–71.4)	95	88.8 (80.9–93.7)	81	78.7 (69.2–85.8)	331	70.8 (66–75.1)
Existence, in the city, of some type of qualification and/or training for PS professionals												
Yes	11	14.8 (8.4–24.9)	9	10.4 (5.3–19.2)	8	11.6 (5.6–22.4)	11	9.5 (4.8–17.8)	11	13.5 (7.6–22.8)	50	11.9 (8.6–16.2)

The city received resources (from the State or Federal governments) intended for structuring PS in the past year												
Yes	23	43.2 (30.4–57)	23	35.8 (24.6–48.7)	37	60.6 (47.2–72.5)	27	42.1 (30.5–54.7)	35	53.9 (41.4–65.9)	145	51.3 (44.8–57.7)
The city spent with the structuring of PS in the past year												
Yes	44	58.5 (47–69.1)	35	44 (32.9–55.8)	50	62.5 (50.5–73.2)	56	59.1 (48.4–69)	35	44.3 (33–56.2)	220	54.8 (49.1–60.5)
The city applied, in the past year, the total value agreed of the corresponding credit of the Basic Component of Pharmaceutical Services												
Yes	37	85.3 (70.8–93.3)	41	73.7 (59.8–84.1)	46	80.5 (67–89.4)	59	92.7 (83–97)	63	90.2 (79.8–95.5)	246	86.4 (81.1–90.4)
The state applied, in the past year, the total value agreed on the corresponding entry of the Basic Component of Pharmaceutical Services^b^												
Yes	25	77 (59.4–88.4)	22	57.2 (40.7–72.3)	27	49.9 (35.1–64.8)	47	86.8 (74.7–93.7)	54	84.2 (72.4–91.6)	175	72.7 (65.7–78.6)

Existence of a permanent bidding committee exclusively for the purchase of medicines in the city												
Yes	43	49 (38.6–59.5)	34	41.4 (31.2–52.4)	26	33.6 (23.5–45.6)	40	36.9 (27.8–46.9)	39	38.1 (28.5–48.7)	182	37.7 (32.6–43)
The Popular Pharmacy Program influences the purchase of medicines by the city^b^												
Yes	54	56.1 (45.8–65.9)	32	33.2 (24.2–43.7)	29	35.9 (25.7–47.5)	61	58.4 (48.6–67.7)	60	61.7 (51.4–71.1)	236	50.9 (45.8–56)
The city purchased medicines to meet judicial demands in the past year^b^												
Yes	71	75.3 (65.3–83.2)	43	47.6 (36.7–58.6)	52	59 (46.3–70.6)	73	69.2 (59.1–77.7)	74	72.4 (62.3–80.7)	313	66.6 (61.3–71.5)

PS: pharmaceutical services

^a^ N value non-weighted

^b^ p < 0.05

Source: PNAUM - Services, 2015.

**Figure 2:**
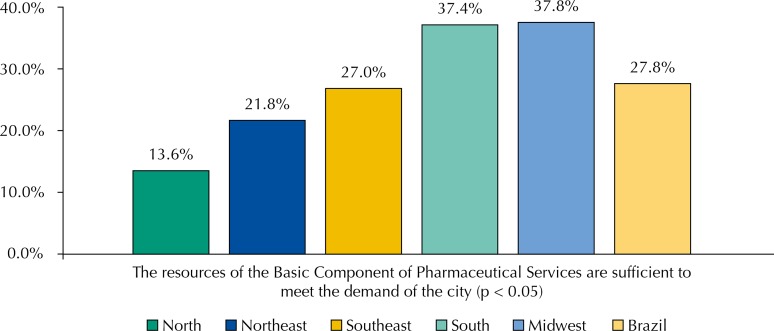
Adequate level of sufficiency of financial resources in the Basic Component of Pharmaceutical Services (CBAF) for meeting the demands, according to those responsible for Pharmaceutical Services, by regions and Brazil. National Survey on Access, Use and Promotion of Rational Use of Medicines – Services, 2015.

Source: PNAUM – Services, 2015.

More than half of the cities received, in the year preceding the survey, resources from the states or federal government aimed at the structuring of PS. Just over 50% of the cities made some expense with PS structuring. As stated by the interviewees, 86.4% of cities applied, in the year before the survey, the total value agreed of the corresponding credit of CBAF, compared with 72.7% of states (Table 1). When asked about the sufficiency of the CBAF’s financial resources to meet the demand of the city, about 37% of those responsible for PS in the South and Midwest regions evaluated it positively, while in the North and Northeast regions these percentages were only 13.6% and 21.8%, respectively (Figure 2).

The existence of a *Comissão Permanente de Licitação* (CPL – Permanent Bidding Committee) – exclusive for the purchase of medicines in the city –, was reported in 37.7% of cities, especially in the Midwest region, with 49%. When asked whether the *Programa Farmácia Popular do Brasil* (PFPB – Brazilian Popular Pharmacy Program) influenced the purchase of medicines carried out by the cities, 50.9% of them have registered affirmative responses. The North region presented the lowest index of interference (33.2%) and the Southeast, the largest one (61.7%). 66.6% of cities have purchased medicines to meet judicial demands in the year preceding the research. The Midwest region stood out with the highest index (75.3%) and the North region with the lowest (47.5%) (Table 1).

**Figure 3:**
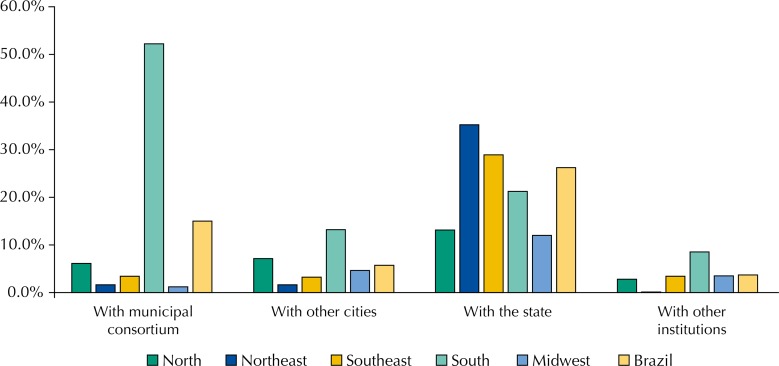
Partners that the cities have established for the purchase of medicines, according to the representatives of municipal Pharmaceutical Services, by regions and Brazil. National Survey on Access, Use and Promotion of Rational Use of Medicines – Services, 2015.

Source: PNAUM – Services, 2015

**Figure 4:**
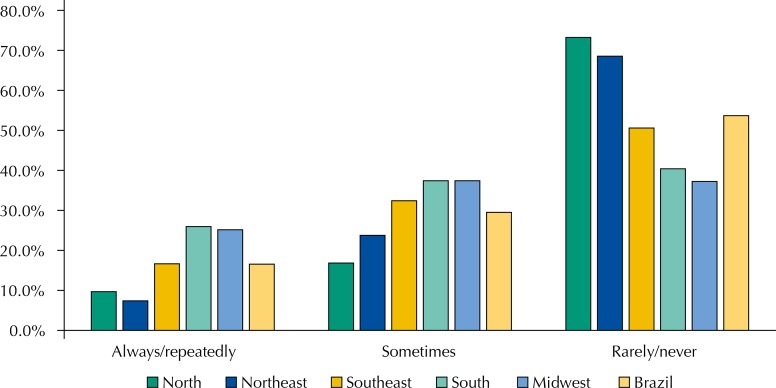
Frequency of purchase of medicines in local pharmacies/drugstores by the cities, according to representatives of Pharmaceutical Services, by regions and Brazil. National Survey on Access, Use and Promotion of Rational Use of Medicines – Services, 2015.

Source: PNAUM – Services, 2015

The association between city and state was the most common partnership for purchasing medicines (26.2%), followed by consortium between cities (15.0%). The South region has adopted the strategy of consortia in 52.2% of the cities (Figure 3). Purchase of medicines carried out in local pharmacies/drugstores was registered in 58.3% of cities, with emphasis for the cities of the North, which had the highest percentage (73.4%) (Figure 4).

## DISCUSSION

Medicines are a vital input in health care^22^, as well as in the process of sustainability of health systems before the volume of financial resources to acquire these items^16^. In a scenario of finite resources and infinite demands, the need for proper management of the resources employed in PS is imperative. In this sense, the results achieved by PNAUM – Services help overcoming this challenge, since they allow one to know and to discuss the factors related to the management of financial resources within the CBAF.

CBAF^12^ is intended for the purchase of medicines and supplies, as specified in Annex I and Annex IV of the *Relação Nacional de Medicamentos Essenciais* (Rename – National List of Essential Medicines), including those related to the diseases and health programs of primary health care, dedicated to meet the individual and collective basic needs of the population^9^. Other items are also part of CBAF: herbal medicines, homeopathic matrices and mother tinctures of Brazilian Homeopathic Pharmacopoeia, and the medicines listed in Group 3 of the PS Specialized Component.

By establishing the sources of funds intended for financing CBAF, the state and municipal secretariats of health determine, in their respective *Comissões Intergestores Bipartite* (CIB – Bipartite Intermanagement Committees), the form of agreement for using the CBAF resources. According to data collected with secretaries of health, 11% of cities agreed to use CBAF resources centrally by the state manager, who took responsibility for acquisition, logistics, and supplies in the cities. Other 31% agreed on the completely decentralized form in municipal management and became responsible for the direct acquisition of the items. However, more than half (54%) chose the partially decentralized mode, which means that the state management of SUS maintained some responsibility for the medicines purchase, even allowing the cities to adhere to their Price Registration Proceedings^6^.

We observed that the PS financial resource management was held autonomously by those responsible for PS in only 19.7% of cities, although most cities (70.8%) have a computerized system for PS management , which allows monitoring and control by the secretariat of health.

Regarding purchases made by cities, 26.2% of them took place in partnership with the state secretariats of health, with emphasis on the Northeast (35.2%) and Southeast (28.9%) regions. We highlight the possibility of states in these regions establishing partnerships with the major public laboratories. The form of the acquisition accomplished by public consortia was adopted by 15% of the cities, especially in the South, where more than 50% of its cities adopted this form of purchase. The *Consórcio Paraná* ^a^ contributed heavily to this result, and the vast majority of cities of this state is associated with it. The North region was the one that reported the lowest rate of partnerships for medicines purchases.

Regardless of the scope of SUS management, all purchases of supplies and medicines should be carried out in agreement with the rules that control purchases, biddings, and contracts of the Public Administration^3^. Almost half of PS managers reported they always, repeatedly, or sometimes purchased medicines in local pharmacies or drugstores. Probably, this practice, which had the biggest instances recorded in the Midwest and South, was in disaccord with current standards.

It is also the managers’ responsibility to register all financial resources related to PS in the Health Plan, in the Annual Schedule, and in the Annual Management Report, as well as to promote the monitoring of these resources, ensure the balance between responsibility and participation in the financing, and provide analyses with information on prices, quantities purchased, and number of patients met^12^.

Still regarding the purchase of medicines, we highlight the practice declared by 35.4% of secretaries: using CBAF resources to situations other than those reported in the agreements. Also, the fact that more than 66% of the cities have bought medicines intended to meet judicial demands is noteworthy. In fact, health litigation has the positive effect of ensuring access to medicines in shortage situations, even filling the lack of care or enabling the incorporation of new health technology. However, it represents a great challenge for SUS, as it puts at risk the achievement of the constitutional principles of the system and causes difficulties for PS management, which may compromise the quality of the use of medicines^17^. It is believed that this phenomenon is aggravated by the lack of trained human resources, which causes weaknesses in the management of the demands of the population, hindering the appropriate guidance to patients in the dispensing units, which ultimately start to operate to a limited extent, as a simple place to deliver medicines^18^. The fact that only 12% of the cities have registered investments in the training of professionals in PS can corroborate such understanding.

A significant consequence of litigation is the disorganization of the health system^b^, especially in the processes of acquisition, since judicial decisions are issued on the basis of urgency, forcing managers to often perform direct acquisitions in pharmacies or drugstores. Certainly, these acquisitions occur at an unfavorable price compared to other forms, which tends to aggravate one of the consequences of this phenomenon: the use of financial resources for the care of individual needs rather than collective needs^19^ ^,^ ^b^.

Still concerning the disorganization of the supplies and medicines purchase, our results showed the interference of PFPB in the acquisition of CBAF medicines. The PFPB was created with the purpose of broadening citizens’ access to essential medicines for the treatment of the most common diseases, by the accreditation of private drugstores, promoting the dispensing of medicines free of charge and/or in co-payment system^4^. The financing of these medicines takes place by the transfer of resources from the Brazilian Ministry of Health to the accredited establishments^13^. In this context, and given a scenario of insufficient resources, municipal secretariats of health may choose not to purchase the list of products offered by the PFPB. It must be considered that the acquisition by cities can be impaired depending on the ability of manufacturers to meet both public and private networks, either by the limitation of production or by the practicality in the process of selling with prices without the discounts conditioned to the sales for the Government. Still, apart from the aspect of organization of processes, the economic aspect must be considered. Recent real world evidence registered that the direct production of PS by the public sector, carried out by a network of its own, is economically more favorable given the outsourcing of PFPB services^8^.

The results related to the funding for structuring and maintaining PS are worrisome. Little more than half of the cities (54.3%) spent in this area, even with the record that 51.3% of cities received some type of financial incentive from the federal or state government. Even lower rates were verified for the investment in qualification and/or professional training for PS workers. This scenario shows a progressive dismantling of the services rendered, in contrast with the fact that, since 2010, it is possible to use up to 15% of the state and municipal resources to finance PS improvements, namely: adjustment of the infrastructure of SUS pharmacies, acquisition of equipment and furniture to support PS actions, and promotion of activities linked to the continuing education and qualification of human resources^14^.

As a way of supporting the national qualification of PS, the Brazilian Ministry of Health instituted, in 2012, the *Programa Nacional de Qualificação da Assistência Farmacêutica no Âmbito do SUS* (QUALIFAR-SUS – National Program for Qualification of Pharmaceutical Services of SUS), prioritizing cities of up to 100,000 inhabitants classified in the range of extreme poverty. The program was organized into four areas: infrastructure, education, information, and care. The funding takes place by the transfer of federal resources destined to acquisition of furniture and equipment required for the structuring of public *Centrais de Abastecimento Farmacêutico* (CAF – Pharmaceutical Supply Centers) and Pharmacies in primary health care, in addition to the maintenance of pharmaceutical services. Until 2016, resources were destined to 1,582 cities, totaling 28% of the Brazilian cities. The investment resource, delivered in a single installment, varies, by population, between R$11.2 thousand and R$24 thousand. The total resource cost is R$ 24 thousand/year, delivered in quarterly form. Although it is an important and necessary program, it shows small impact, either as a source of PS financing or by the number of cities covered – mainly by the low population coverage achieved.

The PS in SUS are funded with municipal, state, and federal budgets, according to the peculiarities of their components: basic, specialized, or strategic. The CBAF is financed with resources from the three levels of government and has application of minimum values, per inhabitant/year, based on the population of each city^12^. The resources are transferred monthly from the federal level to states and from these to the cities, or from the federal government directly to the cities, depending on the type of municipal management agreed. Similarly, there are transfers from the states to the cities, and also there can be the supply of medicines. Besides the transfers from the federal and state governments, one should also consider the resources from the municipal treasury. In addition to the agreed tripartite values, it is responsibility of the Brazilian Ministry of Health to finance, acquire, and distribute insulin, contraceptives, and supplies of the Women’s Health Program, as well as to finance, by annual transfers to states, the medicines purchase in the context of Indigenous Health and in the *Política Nacional de Atenção Integral à Saúde das Pessoas Privadas de Liberdade no Sistema Prisional* (PNAISP – National Policy of Integral Health Care for Persons Deprived of Liberty in the Prison System).

Between 1999 and 2016, a 390% increase was registered in the minimum value *per capita*/year of tripartite financing of CBAF^14^. Similarly, between 2005 and 2009, there was a 61% increase in the total amount spent for purchase of medicines by the three spheres of SUS management^21^. According to our results, 72.7% of the states and 86.4% of the cities applied the total value agreed to finance the CBAF. However, only 9.7% of secretaries of health consider the financial resources intended for the CBAF sufficient to meet the population’s needs. What actually happens is that the values for the CBAF are outdated, especially by the SUS federal manager, responsible for over 50% of the funding. The financial gap, in detriment to the possibility of states and cities having increased values in agreements in the respective CIB, exceeds 50%, when applied to the Broad Consumer Price Index, and becomes even greater if we consider the estimated population growth in the period, more than 12 million people^c^. The discrepancies were exacerbated in 2013, when some medicines of other components migrated to the CBAF, without their respective financial contribution^15^. In this sense, the cities increased their own resources for public health actions and services (ASPS) and for *per capita* expenses with medicines. Between 2010 and 2016, the average percentage of expenses with ASPS jumped from 20.37 to 23.27%^d^, above the 15% recommended by the Federal Constitution. Between 2010 and 2015, the median medicines *per capita expenses* of the cities jumped from R$ 15.71 to R$ 21.04^d^.

Despite the freezing of PS resources, the complete exhaustion of the current model of PS financing takes place, derived from the model of SUS financing. The findings of this research base this understanding as well as the law 141/2012^5^, which regulated, to the financing of the system, the mandatory use of apportionment criteria for the resources of the federal and state governments transferred to other entities, aiming at the progressive reduction of regional disparities. The apportionment of resources linked to ASPS, included there the financing of all primary health care, must consider the health needs of the population, the epidemiological, demographic, socio-economic, and spatial dimensions, as well as the ability to offer health actions and services, and also the combination of the criteria governed by the Law^2^. Additionally, it must employ a method of calculation that considers a general index, built upon the health needs, health care networks, and technical performance of ASPS execution for each of the management levels^20^. As established in law, this is a funding model agreed and standardized for PS, as well as for the SUS, since 2012.

The application of the questionnaire by telephone is a limitation of this research, because it could not allow the respondent access or query to auxiliary material. Also, this is a cross-sectional study, which reflects only the moment of the survey, not enabling the establishment of temporal relationships between the results found.

Finally, the results observed in this study show serious deficiencies in the financing and management of the CBAF financial resources. Among these, we highlight: little concern with the formality in the execution of public resources, either by evidence of acquisition or destination of resources outside of the regulatory standards or by the non-application by the states and cities of their respective corresponding credits; expenses incurred for meeting individual demands to the detriment of collective ones, and; statement of most managers that the resources intended for the CBAF are insufficient to cover needs and demands of the population. The fact that the main financial incentive of PS is below by more than 50% in its value since 2010 corroborates this understanding, as well as the finding, depending on the results obtained and due to legal regulations, of the exhaustion of the current model of PS financing.

In the context of the CBAF, major challenges have not yet been overcome. Considering the multiplicity of responsibilities between the management levels of the SUS to ensure the population’s access to medicines in primary health care, addressing these challenges requires revising the PS financing model, the *Política Nacional de Medicamentos* (PNM – National Drug Policy), and the *Política Nacional de Assistência Farmacêutica* (PNAF – National Policy of Pharmaceutical Services).

## Footnotes

a
*Consórcio Intergestores Paraná Saúde* (Health Intermanagement Consortium of Parana), founded in 1999, constituted in the form of a public consortium, with legal personality under private law, not-for-profit. It gathers 394 cities of the 399 of Paraná and carries out the acquisition of the medicines listed in the Basic Pharmaceutical Services of the state.

b Barroso LR. Da falta de efetividade à judicialização excessiva: direito à saúde, fornecimento gratuito de medicamentos e parâmetros para a atuação judicial. [cited 2017 Jan 10]. Available from: http://www.conjur.com.br/dl/estudobarroso.pdf

c Brazilian Institute of Geography and Statistics. The total population, men and women 2000-2030. Rio de Janeiro: IBGE; 2013 [cited December 11, 2016]. Available from: http://www.ibge.gov.br/home/estatistica/populacao/projecao_da_populacao/2013/.

d Ministry of Health (BR). DATASUS.SIOPS - Information System on Public Budgets in Health. Municipal indicators: 3.2% of own resources applied in health (EC 29) and 2.3% Medicines Expenditure/Total Expenditure. [cited 2017 Jan 10]. Available from: http://siops-asp.datasus.gov.br/CGI/deftohtm.exe?SIOPS/serhist/municipio/mIndicadores.def
